# Flu Vaccination Coverage and Predictors of Non-Vaccination in Military Health Corps Personnel 2016–2017 and 2019–2021

**DOI:** 10.3390/vaccines10030460

**Published:** 2022-03-17

**Authors:** María Julia Ajejas Bazán, Francisco Javier Pérez-Rivas, Julia Wärnberg, Carlos Fuentes Mora, Lucía Elena Ballester Orcal, Jose Manuel Gómez Crespo, Candelas López-López, Silvia Domínguez-Fernández, Milagros Rico-Blázquez, Napoleón Pérez-Farinós

**Affiliations:** 1 Departamento de Enfermería, Facultad de Enfermería, Fisioterapia y Podología, Universidad Complutense de Madrid, 28040 Madrid, Spain; frjperez@ucm.es (F.J.P.-R.); canlopez@ucm.es (C.L.-L.); milarico@ucm.es (M.R.-B.); 2 Academia Central de la Defensa, Escuela Militar de Sanidad, Ministerio de Defensa, 28040 Madrid, Spain; jgomcre@et.mde.es; 3 Grupo de Investigación UCM “Salud Pública-Estilos de Vida, Metodología Enfermera y Cuidados en el Entorno Comunitario”, Departamento de Enfermería, Facultad de Enfermería, Fisioterapia y Podología, Universidad Complutense de Madrid, 28040 Madrid, Spain; jwarnberg@uma.es (J.W.); sildom01@ucm.es (S.D.-F.); napoleon.perez@uma.es (N.P.-F.); 4 Departamento de Enfermería, Facultad de Ciencias de la Salud, Universidad de Málaga, 29071 Málaga, Spain; 5 Instituto de Investigación Biomédica de Málaga (IBIMA), 29071 Málaga, Spain; 6 Departamento de Coordinación y Docencia del Hospital Central de la Defensa, 28040 Madrid, Spain; cfuemor@oc.mde.es; 7 Jefe del Departamento de Enfermedades Infecciosas, Hospital Central de la Defensa, 28040 Madrid, Spain; lbalorc@oc.mde.es; 8 Department of Intensive Care, Hospital Universitario 12 de Octubre, 28041 Madrid, Spain; 9 Grupo de Investigación en Cuidados (InveCuid), Instituto de Investigación Sanitaria Hospital 12 de Octubre (Imas12), 28041 Madrid, Spain; 10 Madrid Salud, Ayuntamiento de Madrid, 28007 Madrid, Spain; 11 Unidad de Investigación de la Gerencia Asistencial de Atención Primaria, Servicio Madrileño de la Salud, 28035 Madrid, Spain; 12 Departamento de Salud Pública y Psiquiatría, Facultad de Medicina, Universidad de Málaga, 29071 Málaga, Spain

**Keywords:** influenza vaccines, vaccination coverage, military personnel, adverse reactions

## Abstract

(1) Background: Vaccination is the most effective intervention to control seasonal influenza morbidity and mortality. The present study aimed to determine the influenza vaccination coverage in the Military Health Corps personnel in the 2020–2021 season, as well as the time trend and the possible influence of the pandemic on coverage, in order to study the reasons that led to the non-vaccination of health professionals and to analyze adverse drug reactions (ADRs). (2) Methods: A descriptive, cross-sectional study was conducted from February to May 2021. All FAS CMS personnel were included. A self-administered questionnaire was sent by e-mail to the selected personnel. (3) Results: Vaccination coverage in the 2016–2017 season was 15.8% (n = 276), in the 2019–2020 season it was 17.41% (n = 424), and in the 2020–2021 season it was 24.22% (n = 590). The percentage of vaccinated men was higher than the percentage of women. In 2019 and 2020 the most vaccinated group was 31–40 years old. Lieutenants had the highest vaccination uptake in 2019 and 2020. The personnel with the highest uptake of vaccines were those in the specialty of nursing in each of 2016, 2019 and 2020, with >30 years of time worked in 2016. In terms of factors leading to refusal of vaccination, the most reported was “not considered a risk group” (23.0%), and the least reported was “avoidance of vaccine administration” (2.2%). Eighty individuals presented adverse reactions after vaccine administration (9.6%). (4) Conclusions: The rate of influenza vaccination among healthcare professionals was lower during the 2020 season compared to the previous season, but was expected to increase in the upcoming 2021 season.

## 1. Introduction

Influenza is one of the most prevalent immunopreventable diseases in developed countries. It is transmitted from person to person, causing seasonal epidemics in winter in countries with temperate climates. Worldwide, it causes between 3 and 5 million cases of severe disease, with 250,000–300,000 deaths per year [1]. In Spain, the number of deaths ranges between 1.61 and 3.37 per 100,000 inhabitants per year [2]. According to data provided by the National Epidemiology Center, influenza had a moderate impact in the 2019–2020 season (less than in the two previous seasons), and would have caused 619,000 confirmed cases of influenza in primary care, 27,700 hospitalizations with confirmed influenza (cumulative rate of hospitalized severe cases with confirmed influenza: 17.7 cases/100,000 inhabitants). Forty-seven percent of the cases were concentrated in those >64 years of age, of which 89.7% involved an A virus, and 9 out of 10 of these were A (H1N1). There were 1800 confirmed cases of influenza admitted to intensive care units and 3900 deaths attributable to influenza [3]. After the onset of the COVID-19 pandemic, influenza circulation changed [1,2], and that this probably had an impact on influenza vaccination coverage rates in different groups [3]. In the 2020–2021 season there have been low levels of influenza activity, which could be due to the social control and distancing measures still in place to control the COVID-19 pandemic, although other factors may have contributed, such as the underreporting of epidemiological and virological data [4]. 

There are communities that are at higher risk for influenza. Currently, due to the COVID-19 pandemic, influenza may facilitate transmission of the disease or worsen its prognosis [1]. 

Vaccination is the most effective intervention to control seasonal influenza morbidity and mortality, as accepted worldwide [5,6].

The Ministry of Health Social Services and Equality of Spain recommends the vaccination of different risk groups. Personnel working in healthcare facilities are part of these groups in which vaccination against influenza is indicated, and their ideal coverage should be 100% in those who do not present contraindications [7,8]. There is no conclusive evidence of the association or not between influenza vaccination and the risk of COVID-19 infection [9,10,11]. The recommendation aims to prevent disease, transmission to patients in whom morbimortality could be increased, avoid the saturation of health centers, and reduce absenteeism in times of high demand for health services [2,12,13]. The percentage estimated as necessary to generate herd immunity and interrupt influenza transmission in health centers is 80% [14]. In contrast, vaccination coverage in this risk group is among the lowest in the world [1,15]. This is not an isolated fact but is common worldwide [16,17]. In a study carried out in eleven European countries, the vaccination percentage in Spain was estimated to be around 25.4% [18,19].

In the Armed Forces (AF), the objectives of vaccination against influenza are similar to those in the civilian sphere, being to protect the health of AF personnel at higher risk of complications in case of influenza, to protect the individual and collective health of personnel deployed in foreign missions, to preserve the capacity to provide services considered essential in the community, and to prevent the transmission of the influenza virus to other persons. Healthcare personnel are considered at risk. This is reflected by the fact that each season the General Inspectorate of Health, with the technical advice of the Institute of Preventive Medicine of the Defense “Captain Ramón y Cajal Medical Doctor” prepares and disseminates recommendations among military personnel [20]. In the current situation, due to the COVID-19 pandemic, an epidemic wave that may temporarily coincide with influenza cannot be ruled out. In this scenario of the possible coincidence of both epidemics, it is considered a priority to prevent the impact of influenza during the autumn-winter. The influenza vaccine administered in the AF complies with World Health Organization (WHO) recommendations for the 2020-2021 season in the northern hemisphere (5). In the AF, influenza vaccination is recommended for personnel in the following groups:

- On overseas deployments:
  ○ units whose deployment in ZO, located in the northern hemisphere, is planned from the beginning of the vaccination campaign until the end of March. In the case of
  ○ deployments to the tropics, vaccination is recommended at any time of the year.
  ○ personnel already deployed in ZO when the vaccination campaign starts.
  ○ alerted personnel, whose availability to deploy to ZO is less than 15 days.
- health personnel.
- personnel assigned or on secondment to the Military Emergency Unit (UME).
- personnel of the Military Educational Centers in which there is a boarding regime.
- risk groups defined by the health authorities

Currently, there is only one study that was conducted by this research team in the 2016–2017 season. The study to be conducted will allow us to know what the vaccination coverage of Military Health Corps (MHC) personnel is and the trends since 2016. There are official data elaborated by the Public Health Directorates of the different autonomous communities of Spain, but they are neither sufficient nor representative of the military personnel. This further justifies the realization and relevance of the present work.

The current group of researchers obtained data on influenza coverage in the 2016–2017 campaign in the same population. The objectives of this work were to determine the 2019–2021 influenza vaccination coverage in the MHC, to describe the trends in coverage since the 2016–2017 campaign and during the 2019–2021 campaign, to analyze coverage according to different factors, to study the reasons that generated the non-vaccination of health professionals, and to analyze the adverse drug reactions (ADRs) as reported by vaccinated professionals associated with the vaccine.

## 2. Materials and Methods

### 2.1. Study

A descriptive, cross-sectional study was conducted from February to May 2021.

### 2.2. Population and Sample

All the personnel of the MHC of the AF were included. This group is dedicated to health care and consisted as of 31 December 2020 of 1800 individuals (732 nurses, 580 physicians, 185 psychologists, 139 pharmacists, 117 veterinarians and 47 dentists). 

### 2.3. Eligibility Criteria

All active personnel assigned to the Army (A), Air Force (AIF), Navy, personnel assigned to units belonging to the Ministry of Defense (MD), those who, despite presenting contraindications, decided to be vaccinated and, finally, those deployed to the area of operations (AO) in the following six months were included. Following the recommendations of the Ministry of Health, Social Services and Equality, personnel who did not perform their activities in a health center were excluded (a health center was defined as an infirmary or first-aid station type structure located in barracks, units, ships and aircraft, ships and hospitals). Likewise, following the recommendations of the AF, we excluded all personnel who were not health personnel by function (those who were dedicated exclusively to management), professionals who presented a contraindication to the administration of the vaccine, or those who were prescribed the vaccine due to a medical condition of risk. 

The initial population was 1750 individuals who complied with the recommendations for the indication of the influenza vaccine. A sample was not selected due to the low expected response.

### 2.4. Variables under Study

The variables studied were sex (male/female), date of birth, organization (Army (A), Air Force (AIF), Navy, Ministry of Defense (MD) and Military Emergency Unit (UME)), fundamental specialty (nursing, medicine, dentistry, pharmacy, veterinary medicine, veterinary medicine, etc.), dentistry, pharmacy, veterinary medicine and psychology), time in the work environment (≤10 years, 11–20 years, 21–30 years and >30 years), employment (lieutenant, captain, commander, lieutenant colonel, colonel and general), cohabitation with persons suffering from chronic diseases, pregnant women and people over 65 years of age (yes/no), reasons for non-vaccination in the personnel who reported not having been vaccinated (doubtful effectiveness of the vaccine, possibility of adverse reactions, fear of the needle, not being considered a risk group, inconvenient schedule and/or work overload and/or forgetfulness, not having been aware of the anti-influenza campaign, having a low probability of getting sick, avoiding the administration of medication, trust in alternative medications and other factors not included in the above), occurrence of adverse reactions (ADRs) (yes/no) [20]. The types of adverse reactions (ADRs), of the different ADRs recorded in the technical file of the influenza vaccine called “frequent” were categorized according to the following variables: pain, headache, swelling, fever, redness, chills, sweating and/or tiredness, ecchymosis and induration, general malaise and myalgia/arthralgia [19] and “having received the influenza vaccine” (yes/no), in 2019–2020 and 2020–2021, as well as the intention to vaccinate in 2021–2022. 

### 2.5. Data Collection Procedure

In the first week of February 2021, the self-administered questionnaire consisting of 21 items was sent by e-mail to HMC staff who met the inclusion criteria. After answering the questionnaire and sending it, it was anonymized in a database prepared to receive the responses. The questionnaire was accompanied by an informative letter about the study. The questionnaire was validated with the first 20% of responses received [21]. Consent forms and willingness to participate in the study were confirmed. A reminder to participate was sent every Monday for two weeks. After this period, the inclusion of questionnaires in the database was finalized.

### 2.6. Statistical Analysis

To calculate the proportion of flu vaccination coverage, the numerator was the total number of vaccinated HMC personnel included in the study and the denominator was all HMC professionals in whom the vaccine was indicated and who met the inclusion criteria. Likewise, the vaccinated personnel were described by absolute and relative frequencies stratified according to sex, fundamental specialty, age, years of work, employment, organization to which they belonged, place where they carried out their activity, and whether they lived with chronic patients, pregnant women or patients >65 years of age. To evaluate whether there was a statistically significant association (*p* < 0.05) between the dependent variable and each of the independent variables, a bivariate analysis was performed using Pearson’s chi-square test. The corresponding CI (95%) were calculated. In addition, the 2016 databases and the one obtained in this study were combined to estimate time trends. A multivariate logistic regression model was used for this purpose. Multivariate logistic regressions were performed using the variables that, in the bivariate variable analysis, were statistically significant (*p* < 0.005) and those that, although not significant, were of interest from a health and epidemiological point of view. The statistical significance was set at 0.05 (two-tailed). The data were tabulated and analyzed using the SPSS 21.0 statistical package for Windows. 

### 2.7. Ethical Aspects

Regarding the ethical aspects of the research, current legislation was respected. The project was presented to the Ethics Committee for Research with Medicines (CEIm) of the General Inspectorate of Defense Health, which certified that the study followed the ethical requirements and postulates. The data were also treated confidentially in accordance with Spanish law (Ley Orgánica 3/2021, de 5 de diciembre, de Protección de Datos de Carácter Personal).

## 3. Results

We started from a population of 1750 individuals, of whom 1120 (64%) were men and 630 (36%) women, with the following main specialties: nurse 711 (40.6%), physician 564 (32.2%), psychologist 180 (10.3%), pharmacist 135 (7.72%), veterinarian 114 (6.5%) and dentist 46 (2.6%), who carried out their activity in UCOs 1442 (82.4%) and hospitals 308 (17.6%). 

The response rate to the questionnaire was 47.54% (n = 832). The mean age of the sample was 43.8 years (SD = 13.6; range 22 to 121 years). Of the total, 52.8% were nurses and 29.1% were physicians, 35.5% had been working for less than 10 years, 33.5% were lieutenants, 66.0% worked in a hospital and 83.9% did not live with people with chronic diseases, nor with pregnant women (97.2%), nor with people over 65 years of age (90.3%). All the results presented in this study refer to the personnel who responded to the questionnaire.

Vaccination coverage in the 2016–2017 season was 15.8% (n = 276), in the 2019–2020 season it was 17.41% (n = 424) and in the 2020–2021 season it was 24.22% (n = 590). Of the total, 24.92% (n = 607) expressed their intention to be vaccinated in the 2021–2022 season.

The percentage of vaccinated men (2016, 77.5%; 2019 66.3% and 2020 61.9%) was higher than the percentage of women (2016, 22.5%; 2019, 33.7% and 2020, 38.1%), finding a statistically significant association (*p* < 0.05). In 2016, the most vaccinated age group was 51–60 years old (47.7%) while in 2019 and 2020 the most vaccinated group was 31–40 years old (27.8% and 23.9%, respectively), with significant association (*p* < 0.05). Likewise, in relation to the army of attachment, those who performed their activity in the OC presented higher vaccination figures, these being of significance only in 2016 (39.8%) and 2019 (47.6%). Lieutenants had the highest vaccination uptake in 2019 (35.1%) and 2020 (36.6%), which were of significance. The personnel with the highest uptake of vaccines were nurses in each of 2016 (44.1%), 2019 (51.2%), and 2020 (54.3%), those with >30 years of time worked in 2016 (43.3%), with less than 10 years in 2019 (38.9%) and 2020 (41. 5%), who lived with chronically ill patients in 2016 (66.7%), and who did not live with them in 2019 (82.1%), who did not live with pregnant women in 2016 (92.1%) and 2019 (96.9%), nor with people older than 65 years in 2016 (83.2%) and in 2019 (88.3), finding in all cases values of *p* < 0.05 (Table 1).

The results of the multivariable analysis to identify predictors of influenza vaccination uptake among the most at-risk subjects are shown in Table 2.

In relation to the factors given for the refusal of vaccination, the most reported was “not being considered a risk group” (23.0%) and the least reported was “avoiding the administration of the vaccine” (2.2%). Women reported not being vaccinated because they were “not a risk group” (54.9%), “because of the possibility of the appearance of AMR” (81.0%), and because they were not aware of the campaign (60.9%). On the contrary, men stated that they had not been vaccinated against influenza because “the schedule was inconvenient, due to work overload or forgetfulness” (65%), “doubtful effectiveness” (84.0%), “low probability of getting sick”, “avoiding the administration of the vaccine” (100.0%), and “high degree of confidence in alternative medicines” (55.6%), with statistical association in all cases (Table 3).

Of all the vaccinated personnel who responded, 80 individuals presented adverse reactions after vaccine administration (9.6%), 100% of them being local. The most frequent were pain and inflammation at the site of administration (23.8% and 22.5%, respectively). All ADRs were more frequent in men than in women, with a statistically significant association (Table 4).

## 4. Discussion

Flu vaccination adherence has increased over the years among CMS staff, reaching the highest coverage during 2020/21, concomitant with the COVID-19 pandemic. Vaccine coverage for the 2019–2021 seasons was calculated, and a time trend study of coverage from the 2016–2017 and 2019–2021 seasons was conducted. In the study previously conducted during the 2016 campaign, a low participation (15.8%) was obtained, and due to this reason, the questionnaire was sent to 1750 individuals out of 1800, having discarded 50 individuals for not meeting the inclusion criteria. At the end of the period for receiving questionnaires, the response rate rose considerably (47.5%), The coverage rate in this study, compared to the first one we did, was considerably higher, although not high enough. In addition, they were higher than the percentage obtained in other studies carried out, where it ranged between 20.1% [1], 38.8% [4] and 36.2% [20]. This change in participation may have been due to the pandemic, which made the staff more sensitive to the situations experienced and the awareness of the seriousness of the prognosis of COVID when combined with influenza, favoring participation in all types of epidemiological studies related to vaccination and COVID-19. It did, however, remain low, perhaps due to the exhaustion of health care personnel.

Likewise, if a sample size calculation had been made with a 5% error rate and a 95% confidence level, the number of individuals should have been 393 as opposed to the 832 that made up our study. The selection through stratified probability sampling by specialty should have included 165 nurses (439 were included), 112 physicians (compared to the 238 included), 43 psychologists (compared to 52 in our study), 34 pharmacists (compared to 41), 31 veterinarians (compared to the 23 in our study) and eight dentists (compared to 25). In our case, nurses were overrepresented with respect to the rest; perhaps they were more sensitized due to the different functions they adopted during the pandemic, as they directed the epidemiological surveillance and the study of contacts by orienting the tracers, prescribed and administered vaccines against COVID and performed PCR and antigen testing, as well as provided clinical assistance in the ICU of the two military hospitals [22].

In relation to influenza vaccination coverage in Spain, the objectives for the 2020–2021 season established by the Ministry of Health were to achieve or exceed vaccination coverage of 75% in healthcare personnel [23,24]. Each autonomous community (CCAA) annually notifies the Ministry of Health, Social Services and Equality (MSSSI) of the coverage achieved against influenza in professionals. The information obtained is uneven and variable, although it is below what is desirable. In our study the coverage rate obtained was 17.41% in the 2019–2020 season, and in the 2020–2021 season it was 24.22%. These figures are quite a bit higher than previous records (2016–2017 season, 6.9%) of influenza vaccination among healthcare workers in FAS. Perhaps the finding that COVID-19 and influenza virus infection increased the risk of death significantly [14] may have stimulated positive attitudes of healthcare workers towards influenza vaccination. Despite there being a significant increase, the rates were still lower than those found in other similar studies such as the one conducted in Greece, where coverage was 74.0% [25], or the one conducted in Canada where coverage was 74% (2018–2019) and 72. 0% (2019–2020) [26], or the 30.6% obtained in a study in Italy [27] or studies from the United Kingdom, where they found that the COVID-19 pandemic motivated a higher uptake of influenza vaccination in 2020–2021 in subjects who were not usually vaccinated [28]. The figure in our study is far from the 75–80% recommended by the WHO and the European Commission (EC) [24]. Perhaps CMS staff were less responsive to vaccination promotion campaigns and messages [2]. Although the numbers were not as desired, reported cases of influenza barely reached 1% during the months of December to February in the FAS and in Europe [14,15]. Factors that may have influenced this were the use of masks, social distancing and a significant increase in influenza coverage. A relative increase in influenza immunization intention was observed in the 2021–2022 campaign. This dynamic pattern was also observed during the A (H1N1) pandemic [16] and the current COVID-19 pandemic [17]. However, it is unclear whether this increase in intention will translate into increased coverage in the next campaign.

In relation to sex, the percentage of vaccinated men was higher in the three seasons, although with a slight increase in women from the 2016–2017 season (22.5%) to the 2020–2021 season (38.1%). Similar data were obtained in a study conducted among healthcare personnel in an Italian center [18]. On the contrary, a study conducted in Saudi Arabia showed that women were more compliant than their male counterparts. The reasons that may have caused men to vaccinate more than women may be due to chance, although there is a statistical association, since there is no theoretical basis a priori to support this result [29]. 

Similarly, during the 2016–2017 season as age and number of years worked increased, the percentage of vaccinated individuals increased (*p* < 0.05), but it was observed how in the 2019–2020 campaign the percentage of younger personnel, with less time worked and those with the job of lieutenant increased, and even surpassed the older ones in the 2020-2021 campaign. It suggests a change in the attitude of CMS staff towards influenza vaccination, especially among younger people, perhaps because the current curriculum of health sciences degrees gives greater importance to prevention against the disease [1]. The greatest progress in adherence to vaccination during the pandemic has been observed among younger women (i.e., 20–30 years of age). Similar results were found in a study done in Italy [18]. The results were, however, different in some other studies, such as one done in Saudi Arabia where the highest adherence was found in the age group of those over 40 years [15], and another done in Italy where older nurses and physicians showed higher adherence data [19].

In relation to each specialty individually, the one that presented the highest vaccination percentages was nursing, which also increased its percentage from the first campaign to the current one (44.1% vs. 54.3%, respectively). It seems that it is the most proactive specialty, having the highest rate of influenza vaccination, perhaps because they are more aware, have a greater perception of risk and are the youngest group. In the coming years, it will be important to focus vaccination promotion efforts on new targets such as physicians, pharmacists, psychologists, veterinarians and dentists, who continue to show the lowest adherence. In other studies the results were different; in one conducted in Italy, the most vaccinated specialty discipline was medicine [30].

CMS personnel assigned to the Ministry of Defense and the Army were the most vaccinated, and their numbers increased during the three campaigns (*p* < 0.05). This could be due to the fact that the CMS professionals assigned to these Armies carried out activities during the pandemic that entailed a higher risk of transmission of the disease, as they were in the hospital and this may have generated the need for protection against influenza. With respect to the percentage of CMS professionals who lived with people with chronic diseases, pregnant women or those aged ≥65 years, there was a significant decrease in the percentage of vaccination compared to those who did not live with people from those groups. Our results were not consistent with those of other studies [7,31]. This is perhaps because the personnel who were vaccinated in our study were younger and the coexistence with these people was lower, generating a feeling of protection towards them.

In the multivariate analysis of the last two seasons, being a man belonging to the specialty of nursing, in the age range of 31–40 years, assigned to the OC, with the job of lieutenant and with less than 10 years of working time was associated with a higher probability of influenza vaccination in the last two campaigns. The most relevant findings with respect to the previous study is that the acceptance of vaccination in professionals over 60 years of age and with more than 30 years of service is unacceptably lower and also decreases over time [32,33,34].

To our knowledge, this is the first study to explore factors affecting influenza vaccination coverage during the COVID-19 pandemic in Spanish FAS CMS personnel by assessing how the pandemic has changed attitudes toward influenza vaccination. Our study shows the contribution of the COVID-19 pandemic to increased adherence to influenza vaccination among CMS personnel. However, more interventions should be made to achieve higher vaccination coverage. Some possible strategies to improve vaccination coverage that could be implemented could include providing incentives to healthcare personnel to monitor and encourage vaccine uptake or reducing the age at which the recommendation becomes universal, in addition to encouraging delivery in non-traditional settings [21,22]. It should be noted that it is likely that vaccination against COVID-19 will be necessary in the next few years, and dual vaccination could be a problem in the future unless bivalent vaccines for influenza and COVID-19 are designed.

The main reason given by CMS staff for not being vaccinated was “not being a risk group” (23.0%), followed by “other factors” (17.1%). Similar results were obtained in other studies, where the first reason was “low perceived risk of getting sick” followed by “avoidance of medication”, “confidence in their immune system” or “fear of AMR.” [35,36]. One of the reasons that may lead CMS staff to believe that they are “not a risk group” is that they work with healthy staff whose age range is between 19–61 years. Likewise, they are not aware of the need to protect themselves from sick patients. A higher percentage of women reported “the possibility of the appearance of AMR” as a factor for not being predisposed to vaccination, compared to men who reported “doubtful effectiveness” and “low probability of getting sick”, with a statistically significant association, and no apparent reason was found for these differences [37].

Vaccinated CMS personnel reported an ADR rate of 2.5%. None suffered serious reactions or allergic reactions, and the others were mainly pain and swelling at the site of administration. They were more frequent in men versus women for any type of reaction (*p* < 0.05). In some studies conducted in Spain, the percentage of ADRs was similar [38,39,40]. In relation to serious ADRs, other studies have found similar results, with no serious ADRs [41], reinforcing the safety profile of the vaccine.

The limitations of this study include nonresponse bias, which is common in this type of research [42], and selection bias, since the professionals who responded to the questionnaire could have a higher vaccination coverage than nonresponders due to their greater awareness and concern [43]. In addition, self-reported measures of vaccination coverage were used, being subject to recall bias. Although there may be an overestimation, this way of obtaining information may be the only effective and feasible way to collect data [44,45].

Following the results obtained, measures aimed at increasing vaccination coverage rates among CMS professionals will be implemented in future seasons. The results of this intervention will also be evaluated in the next season.

## 5. Conclusions

Flu vaccination coverage of CMS personnel has increased significantly with respect to previous campaigns, being higher in men belonging to the specialty of nursing in the age range of 31–40 years in the last two campaigns and attached to the OC and with the job of lieutenant. The greatest progress in adherence to vaccination during the pandemic has been observed among younger women (i.e., 20–30 years of age). The specialty that presented the highest vaccination percentages was nursing, and personnel assigned to the Ministry of Defense and the Army were the most vaccinated.

Being a man working in the specialty of nursing, in the age range of 31–40 years, assigned to the OC, with the job of lieutenant and with less than 10 years of working time was associated with a higher probability of influenza vaccination in the last two campaigns.

The main factor motivating the non-vaccination of the personnel was “not being a risk group”, followed by “other factors”.

The most frequent adverse reactions were pain and swelling at the site of administration, and this was more frequent in men. No serious reactions or allergic reactions were reported. 

The uptake rate of influenza vaccination among healthcare professionals was lower during the 2020 season compared to the previous season, but is expected to increase in the upcoming 2021 season. Nevertheless, the results are encouraging and warrant an increased willingness of healthcare professionals to receive influenza vaccination in the upcoming 2021 season.

## Figures and Tables

**Table 1:** Influenza vaccination coverage against seasonal influenza in CMS according year (2016, 2019, 2020) and study variables.

		Vaccinated 2016–2017 n (%)	*p*-Value	Vaccinated 2019–2020 n (%)	*p*-Value	Vaccinated 2020–2021 n (%)	*p*-Value
Sex	Male	93 (77.5)	*p* < 0.005	281 (66.3)	*p* < 0.005	365 (61.9)	0.028
	Female	27 (22.5)		143 (33.7)		225 (38.1)	
Age groups	22–30	7 (9.1)	*p* < 0.005	69 (16.3)	*p* < 0.005	137 (23.2)	0.002
	31–40	14 (15.0)		118 (27.8)		141 (23.9)	
	41–50	24 (26.4)		98 (23.1)		125 (21.2)	
	51–60	75 (47.7)		91 (21.5)		131 (22.2)	
	>60	2 (1.8)		48 (11.3)		56 (9.5)	
Army	Ministry of Defense	40 (39.8)	*p* < 0.005	202 (47.6)	*p* < 0.005	269 (45.6)	0.140
	Army	24 (24.8)		120 (28.3)		200 (33.9)	
	Navy	14 (10.6)		43 (10.1)		55 (9.3)	
	Air Force	24 (24.8)		36 (8.5)		43 (7.3)	
	Military Emergency Unit	0 (0.0)		23 (5.4)		23 (3.9)	
Military grades	Lieutenant			149 (35.1)	*p* < 0.005	216 (36.6)	0.022
	Captain			132 (31.1)		181 (30.7)	
	Commander			59 (13.9)		75 (12.7)	
	Lieutenant Colonel			55 (13.0)		89 (15.1)	
	Colonel			28 (6.6)		28 (4.7)	
	General			1 (0.2)		1 (0.2)	
Specialty in health	Nurse	53 (44.1)	*p* < 0.005	215 (51.2)	*p* < 0.005	318 (54.3)	*p* < 0.005
	Medicine	49 (40.8)		147 (35.0)		188 (32.1)	
	Pharmacy	8 (6.6)		15 (3.6)		18 (3.1)	
	Psychology	9 (7.7)		32 (7.6)		38 (6.5)	
	Veterinary	0 (0.0)		5 (1.2)		9 (1.5)	
	Dentistry	1 (0.8)		6 (1.4)		15 (2.6)	
Time worked	≤10	10 (8.4)	*p* < 0.005	165 (38.9)	*p* < 0.005	243 (41.5)	*p* < 0.005
	11–20	23 (19.2)		64 (15.1)		90 (15.4)	
	21–30	35 (29.1)		101 (23.8)		152 (25.9)	
	>30	52 (43.3)		94 (22.2)		101 (17.2)	
Place of work	Units/Centers/Organizations	96 (80.0)	0.782	320 (75.5)	0.002	470 (79.7)	0.599
	Hospital	24 (20.0)		104 (24.5)		120 (20.3)	
Do you live with chronically ill people?	Yes	20 (66.7)	*p* < 0.005	76 (17.9)	*p* < 0.005	98 (16.6)	0.181
	No	93 (37.8)		348 (82.1)		492 (83.4)	
Do you live with pregnant women?	Yes	9 (7.9)	*p* < 0.005	13 (3.1)	0.002	13 (2.2)	0.083
	No	104 (92.1)		411 (96.9)		577 (97.8)	
Do you live with people over 65 years old?	Yes	19 (16.8)	*p* < 0.005	49 (11.7)	0.001	52 (8.9)	0.108
	No	94 (83.2)		371 (88.3)		534 (91.1)	

**Table 2:** Predictors of influenza vaccination uptake high risk subjects.

		Vaccinated 2016–2017 OR (CI 95%)	Vaccinated 2019–2020 OR (CI 95%)	Vaccinated 2020–2021 OR (CI 95%)
Sex	Male	2.58 (1.84–3.63)	1.86 (1.41–2.44)	1.67 (0.95–2.95)
	Female	1	1	1
Age groups	22–30	2.31 (0.76–7.04)	4.56 (2.54–8.21)	4.00 (1.31–12.19)
	31–40	3.54 (2.05–6.10)	10.89 (3.70–32.10)	4.56 (2.54–8.21)
	41–50	3.67 (2.77–4.87)	10.09 (6.63–15.34)	3.66 (2.75–4.46)
	51–60	5.62 (3.85–8.21)	6.65 (4.90–9.02)	3.51 (1.98–6.03)
	>60	1	1	1
Army	Ministry of Defense	2.58 (0.89–7.44)	2.08 (1.33–3.20)	
	Army	2.16 (1.18–3.95)	1.64 (0.86–3.12)	
	Navy	2.02 (1.30–3.13)	1.58 (0.85–2.94)	
	Air Force	1.58 (0.85–2.94)	1.27 (0.40–4.00)	
	Military Emergency Unit	1	1	
Military grades	Lieutenant		1.43 (1.18–1.76)	1.56 (1.06–1.68)
	Captain		1.39 (1.12–1.95)	1.42 (1.21–1.86))
	Commander		1.31 (1.16–1.45)	1.37 (1.17–1.38)
	Lieutenant Colonel		1.30 (1.07–1.55)	1.29 (1.10–1.49)
	Colonel		1.29 (1.07–1.52)	1.22 (1.15–1.39)
	General		1	1
Specialty in health	Nurse	3.65 (2.76–4.85)	3.72 (2.69–4.92)	3.95 (2.15–5.15)
	Medicine	3.56 (2.06–6.10))	3.68 (2.13–5.58)	3.75 (2.14–6.20))
	Pharmacy	2.25 (1.02–4.95)	2.32 (1.15–5.05)	2.47 (1.02–4.95)
	Psychology	2.17 (1.19–3.94)	2.26 (1.08–4.14)	2.39 (1.32–4.04)
	Dentistry	2.02 (1.30–3.14)	2.13(1.12–3.26)	2.19 (1.67–4.04)
	Veterinary	1	1	1
Time worked	≤10		1.43 (1.18–1.82)	1.52 (1.06–1.92)
	11–20		1.39 (1.09–1.88)	1.48 (1.16–1.65)
	21–30		1.31 (1.14–1.44)	1.33 (1.22–1.34)
	>30		1	1

**Table 3:** Non-vaccination factors.

				Sex				
		Total		Male		Female		*p*-Value
		n (%)	CI 95%	n (%)	CI 95%	n (%)	CI 95%	
Non-vaccination factors	No risk group	51 (23.0)	(17.5–28.5)	23 (45.1)	(38.6–51.6)	28 (54.9)	(48.4–61.4)	*p* < 0.005
	Possibility of occurrence of AMR	21 (9.5)	(5.6–13.4)	4 (19.0)	(13.8–24.2)	17 (81.0)	(75.8–86.2)	
	Inconvenient schedule, overload, forgetfulness	20 (9.0)	(5.2–12.8)	13 (65.0)	(58.7–71.3)	7 (35.0)	(28.7–41.3)	
	Doubtful effectiveness	25 (11.3)	(7.1–15.5)	21 (84.0)	(79.2–88.8)	4 (16.0)	(11.2–20.8)	
	Low probability of getting sick	30 (13.5)	(9.0–18.0)	24 (80.0)	(74.7–85.3)	6 (20.0)	(14.7–25.3)	
	No knowledge of campaign	23 (10.3)	(6.3–14.3)	9 (39.1)	(32.7–45.5)	14 (60.9)	(54.5–67.3)	
	Avoid vaccine administration	5 (2.2)	(0.3–4.1)	5 (100.0)	(100.0–100.0)	0 (0.0)	(0.0–0.0)	
	Reliance on alternative medicines	9 (4.1)	(1.5–6.7)	5 (55.6)	(49.1–62.1)	4 (44.4)	(37.9–50.9)	
	Other factors	38 (17.1)	(12.1–22.1)	17 (44.7)	(38.2–51.2)	21 (55.3)	(48.8–61.8)	
Total		222 (100.0)	(100.0–100.0)	121 (54.5)	(47.9–61.1)	101 (45.5)	(38.1–52.9)	

**Table 4:** Types of adverse reactions.

				Sex				
		Total n (%)	CI 95%	Male n (%)	CI 95%	Female n (%)	CI 95%	*p*-Value
Types of adverse reactions	Induration	12 (15.0)	(7.2–22.8)	8 (66.7)	(56.4–77.0)	4 (33.3)	(23.0–43.6)	*p* < 0.005
	Erythema	9 (11.3)	(4.4–18.2)	9 (100.0)	(100.0–100.0)	0 (0.0)	(0.0–0.0)	
	Myalgia_arthralgia	4 (5.0)	(0.2–9.8)	4 (100.0)	(100.0–100.0)	0 (0.0)	(0.0–0.0)	
	Inflammatión	18 (22.5)	(13.3–31.7)	13 (72.2)	(62.4–82.0)	5 (27.8)	(18.0–37.6)	
	Pain	19 (23.8)	(14.1–33.5)	10 (52.6)	(41.7–63.5)	9 (47.4)	(36.5–58.3)	
	Fever	9 (11.2)	(4.3–18.1)	5 (55.6)	(44.7–66.5)	4 (44.4)	(33.5–55.3)	
	Other	9 (11.2)	(4.3–18.1)	0 (0.0)	(0.0–0.0)	9 (100)	(100.0–100.0)	
Total (n = 832)		80 (100.0)	(100.0–100.0)	49 (61.2)	(50.5–71.9)	31 (38.8)	(28.1–49.5)	

## Data Availability

Not applicable.
